# Laboratory and Full-Scale Tests of Modern Chimney Casings Based on Lightweight Perlite Concrete with Hydrophobic Admixtures

**DOI:** 10.3390/ma18143398

**Published:** 2025-07-20

**Authors:** Arkadiusz Mordak, Krzysztof Drozdzol, Damian Beben, Pawel Jarzynski

**Affiliations:** 1 Faculty of Civil Engineering and Architecture, Opole University of Technology, 45-061 Opole, Poland; a.mordak@po.edu.pl (A.M.); k.drozdzol@po.edu.pl (K.D.); 2 Jawar Sp. z o.o., 06-400 Ciechanow, Poland; pawel.jarzynski@jawar.com.pl

**Keywords:** chimney block, durability of structures, expanded perlite, perlite concrete, water absorption, hydrophobisation

## Abstract

Currently, chimney technology is looking for new materials with improved thermal insulation properties and, at the same time, adequate durability. The use of concretes based on lightweight aggregates, such as expanded perlite, is capable of meeting such a challenge, provided that the composition of the concrete mixes is appropriately modified. The main research challenge when designing chimney system casing elements lies in ensuring adequate resistance to moisture penetration (maximum water absorption of 25%), while achieving the lowest possible bulk density (below 1000 kg/m^3^), sufficient compressive strength (minimum 3.5 MPa), and capillary water uptake not exceeding 0.6%. In the present research, laboratory tests were conducted to improve the fundamental technical properties of lightweight perlite-based concrete to meet the aforementioned requirements. Laboratory tests of perlite concrete were carried out by adding eight chemical admixtures with a hydrophobic effect and the obtained results were compared with a reference concrete (without admixtures). However, the positive results obtained under laboratory conditions were not confirmed under actual production conditions. Therefore, further tests were conducted on chimney casings taken directly from the production line. Subsequent chemical admixtures with a hydrophobic effect, based on silane/siloxane water emulsions, were applied to determine the concrete mix’s optimal composition. The results of the tests carried out on perlite concrete chimney casings from the production line confirm the effectiveness of the applied chemical admixtures with a hydrophobic effect in improving the moisture resistance. This was further supported by the outcomes of the so-called ‘drop test’ and capillary uptake test, with the suitable bulk density and compressive strength being maintained.

## 1. Introduction

Currently, the literature describes, and the market offers, chimney flues whose claddings are made of steel and concrete [1,2,3,4,5,6]. Steel chimneys are used in existing buildings, whereas concrete chimneys, which are the subject of this study, are applied in newly constructed buildings [7]. Currently, the primary focus in developing concrete chimney technology is on achieving high thermal insulation, low weight, and durability (including resistance to aggressive environmental conditions) [1,8,9].

The answer to the challenge of durability, including compressive strength and water absorption, may be the hydrophobisation of chimney blocks at the production stage. This treatment may protect against moisture when the chimney blocks are exposed to moisture effects from atmospheric precipitation and water vapour condensation inside the chimney. This issue is particularly relevant for innovative chimney systems, in which the air layer is used to supply combustion air. Penetrating moisture has a critical impact on material weakness, leading to the formation of cracks caused by the cyclic freezing and thawing of water within the pores of the block. Moreover, dry chimney blocks exhibit better insulation properties than those saturated with water [10,11]. Moisture in concrete chimney blocks increases their thermal conductivity, deteriorating the chimney’s insulating performance. This negatively affects the draft and compromises the safety of flammable materials located near the chimney [6].

Studies [12,13,14,15] demonstrated that the hydrophobisation of concrete products affects their durability and effectively protects against the impact of weather conditions. This justifies carrying out tests to check the properties of hydrophobised chimney blocks. Adding chemical admixtures with a hydrophobic effect to concrete reduces water permeability under increased pressure and the material’s water absorption. These admixtures counteract capillary rise, thereby hindering the penetration and flow of water within the concrete [16].

In contrast, sealing admixtures are used to improve the watertightness and reduce the water absorption of concrete. This leads to a significant increase in the durability of the concrete. Increased concrete tightness resulting from the action of chemical admixtures can be achieved in several ways:

- Filling the pores with mineral powders (which may increase the density of the concrete) [17];
- Introducing substances that react with Ca(OH)_2_ (a cement hydration product), resulting in the formation of poorly soluble chemical compounds that fill the pores [18];
- Reducing wettability—hydrophobisation—hindering the penetration of aggressive agents [19];
- Reducing the amount of mixing water—a beneficial decrease in the water-to-cement (*w*/*c*) ratio [20].

Admixtures that enhance watertightness may operate through various mechanisms, but their primary effect is to impart hydrophobic properties to concrete, which means increasing the contact angle between the walls of capillary pores and water, so that the water is ‘repelled’ from the pores [21].

It should be noted that the sealing effect and the reduction in water permeability can also be achieved through the use of finely ground mineral additives, such as silica dust, stone powder, bentonite, ground pozzolans, and fly ash. However, using fine fractions increases water demand, necessitating a higher amount of mixing water to achieve the desired workability. This has an adverse effect on the strength parameters of concrete [16,22,23]; therefore, such additives were not used in the present study.

The effect of admixtures based on silanes/siloxanes used in the concrete mix becomes noticeable after the completion of the setting and hardening (curing) process of the concrete under normal conditions. There is a significant improvement in resistance to capillary water and moisture absorption [24,25,26]. There are also admixtures based on amino alcohols, which combine the effects of sealing admixtures and those that induce crystallisation within the capillaries of concrete [27]. They are used to reduce concrete permeability and can seal cracks.

In study [28], mortars with expanded clay aggregate were investigated. Chemical admixtures with a hydrophobic effect based on organosilicon compounds were used. Mass hydrophobisation was achieved by using hydrophobic admixtures, while surface hydrophobisation was carried out using a methyl silicone resin solution. The test results indicate that surface hydrophobisation becomes ineffective after 14 days, and the longer the material remains in contact with water, the lower the effectiveness of the impregnation. In contrast, mass hydrophobisation slightly reduced the porosity of the mortar, resulting in a 27% increase in its compressive strength. The aim of the study [29] was to evaluate the effect of using hydrophobic agents based on organosilicon compounds to protect lightweight expanded clay blocks containing sewage sludge against moisture. Two agents for surface hydrophobisation were applied, based on methyl silicone resin in potassium hydroxide and organic solvent-based methyl silicone resin. The second admixture proved to be more effective. It was shown that contact angles decreased over time for the hydrophobising substances used (indicating a reduction in their effectiveness), and the extent of these changes depended on the type of agent applied.

Surface hydrophobisation products are available on the market [30,31,32,33,34], which are most commonly based on oligomeric siloxanes, acrylic resins, or silicone dispersions in an organic solvent. They are characterised by low molecular weight, resulting in high capillary penetration capability, enhancing their effectiveness.

Another challenge in this study is obtaining a low-density chimney block with adequate thermal insulation. Therefore, it was decided to use expanded perlite (EP), which is characterised by low bulk density and is widely used as an aggregate for producing lightweight concrete and as a material for thermal and acoustic insulation [35]. EP also enhances the fire resistance of building partitions. On the other hand, due to its tendency to absorb water, EP contributes to the deterioration of concrete’s physical and mechanical properties [36]. This has led to the search for methods to protect perlite-based lightweight concrete against water absorption, such as coating EP with polymer layers [37].

In recent years, there has been a growing need to develop building components capable of improving the thermal insulation of building envelope elements. This is related to the need to improve the thermal efficiency of buildings, which significantly impacts the construction sector’s environmental performance, especially in the context of the growing demand for a balance between the natural environment and civil engineering. Expanded perlite (EP) is one of the materials that has been considered in the development of modern construction applications aimed at improving the thermal efficiency of buildings [38,39,40,41], including passive [10].

Research is also being conducted on building materials demonstrating perlite’s benefits. According to studies by the Perlite Institute (International Association of Perlite), walls made of formwork blocks from lightweight concrete, when filled with EP, show an improvement (reduction) in the heat transfer coefficient ranging from nearly 200% to over 300% (depending on wall thickness) compared to unfilled walls [42]. Research has also shown that an EP content in concrete exceeding 20% classifies the concrete as an insulating material due to a significant loss in strength [43].

Concluding, it can be stated that EP has not yet been widely used for the production of chimney blocks, which is also reflected in the lack of publications in this area. A research gap was identified in using and testing lightweight perlite concrete in chimney technology. Therefore, it is essential to investigate and understand the main properties of such a material to be used in energy-efficient construction. The present study aimed to improve the durability of prefabricated chimney casings made of lightweight perlite-based concrete. To achieve a positive outcome, it was necessary to reduce moisture ingress into the chimney casing structure, which results in decreased degradation of the chimney block structure. The research aimed to reduce the water absorption of perlite concrete while maintaining adequate strength parameters.

It was planned that the developed perlite-based chimney casing would have properties comparable to those of chimney blocks currently available on the market, as shown in Table 1, and not inferior in terms of functionality or durability. It would meet the following criteria: material density below 1000 kg/m^3^, compressive strength of at least 3.5 MPa, maximum water absorption of 25%, and maximum capillary uptake of 0.6%. The assumption of the lowest possible density is related to the reduction in anticipated transportation costs of chimney blocks and is aligned with the principles of sustainable development.

## 2. Materials and Procedure of Testing

### 2.1. Materials in Laboratory Tests

Laboratory tests of perlite concrete based on various mixes (different component proportions/different chemical admixtures) were conducted in the initial preparatory phase before the commencement of full-scale trials on the production line. Based on previously obtained results, it was decided that water absorption tests of concrete samples using different chemical admixtures were necessary. The reference mix (R0) for testing the properties of perlite concrete was the composition presented in Table 2. This was the mix used on the production line by a leading manufacturer of perlite concrete blocks.

A superplasticiser based on non-sulfonated acrylic polymers (S1) was used to prepare the mix. Tests were conducted using seven chemical admixtures with a hydrophobic effect, each with different mechanisms of action. As a result, subsequent mix designs were designated as R1–R9:

- R1—reference sample—without hydrophobic admixture;
- R2—with admixture A1 (1%) based on alkoxysilanes (the main component is a non-ionic alkoxysilane emulsion);
- R3—with A2 (1%) based on 2-octylisothiazolone;
- R4—with A3 (1%) based on calcium stearate;
- R5—with A4 (1%) based on silanes;
- R6—with A5 (1%) based on silicone;
- R7—with A6 (1%) based on alkylalkoxysilane;
- R8—with A7 (1%) based on modified organic salts;
- R9—with A7 (1%) based on modified organic salts, with prior moistening of the aggregate.

The expanded perlite used as aggregate for preparing the perlite concrete samples, both with and without various admixtures, was subjected to bulk density testing (several test series were conducted), yielding an average value of 100 kg/m^3^.

### 2.2. Materials in Production Line Tests

In the following research stage, modifications were introduced to the base mix R0 due to the need to adapt the mixtures to the conditions on the production line. First and foremost, the amount of mixing water was increased to 160 L per batch, which necessitated testing the compressive strength of the finished perlite concrete blocks. Additionally, new chemical admixtures with a hydrophobic effect (A8–A11, in amounts ranging from 0.5% to 5% of the cement mass) were introduced:

- A8—admixture based on a silane/siloxane water emulsion (pH 7.0–9.0);
- A9—based on a silane/siloxane water emulsion (pH 6.0–8.0);
- A10—based on silanes (pH 7.0–9.0);
- A11—based on calcium stearate.

This created the need to determine appropriate admixture dosages, which led to the design of mix formulations R16–R24 (Table 3), from which test samples were prepared.

In the subsequent tests (conducted on blocks and samples cut from finished chimney blocks), mix formulations containing chemical admixture A11 were excluded due to the least favourable results obtained in the so-called “water drop test” and capillary uptake test carried out on R16–R24 mixes from the production line. Due to the requirement to achieve hydrophobisation of the perlite concrete mix, it was additionally decided to test the performance of plasticising admixtures A12, based on a polymer solution, and A13, based on an aqueous solution of modified resins.

Because this stage of the research was conducted on finished products and samples cut from them, new mix designations were introduced: P0–P5 (to distinguish them from the previous ones, in which samples were formed from the perlite concrete mix). These are referred to as Production line samples (Table 4). At the same time, due to favourable differences in the obtained test results for higher dosages of chemical admixtures with a hydrophobic effect, and taking into account economic considerations, it was decided to use admixtures A8–A10 in the amount of 2% of the cement mass (Table 4). Additionally, during the concrete mix production, the moisture content was adjusted by regulating the amount of mixing water to achieve the desired consistency.

Tests of water absorption by weight, density, and compressive strength of perlite concrete were conducted on samples prepared in the laboratory and formed from the mix taken from the production line, as well as on blocks and samples cut from finished chimney blocks. Each test was performed on a minimum of six samples.

### 2.3. Test Procedures

*Compressive Strength Test.* For these tests, cubes of 100 × 100 × 100 mm were prepared, made of a perlite concrete mixture and stored by PN-EN 12390-2 [47]. The samples needed to have flat and parallel front surfaces. The test was performed at a temperature of 20 ± 2 °C. The perlite concrete samples were tested after 28 days of curing. The sample was placed centrally in the strength press. The applied load increased smoothly and without shocks at a constant stress growth rate of 0.6 ± 0.2 MPa/s (in the range of 5–90% of the predicted strength). The perlite concrete sample was loaded until failure, and the maximum failure force (F) was recorded. The compressive strength of the samples *f*_cm_ [MPa] was calculated according to the Equation (1):

(1) $$f_{cm}=\frac{F}{A}\;$$

where *F*—breaking force (N), *A*—cross-sectional area of the sample (mm^2^).

*Density Test.* This test was performed on perlite concrete samples of regular shape, i.e., cubes of dimensions 100 × 100 × 100 mm, prepared by [47]. The samples were clean and dry on the surface and dried to a constant mass. The sample volume was calculated based on its measured dimensions (with an accuracy of 1 mm). The perlite concrete sample was weighed with an accuracy of 10 g after drying at 105 ± 5 °C to a constant mass. The density of the perlite concrete samples *ρ* (kg/m^3^) was calculated using Equation (2):

(2) $$\rho =\frac{m}{V}$$

where *m*—sample mass (kg), *V*—sample volume (m^3^).

*Perlite Concrete Water Absorption Test*. For these tests, samples were prepared as cubes with dimensions of 100 × 100 × 100 mm. Then, after hardening, the samples were dried to a constant mass at a temperature of 105 ± 5 °C (weighing was carried out every 24 h until the mass change was less than 0.2%). The dried samples were wholly immersed in water at 20 ± 2 °C. The initial saturation of the samples lasted for 72 h, with the water constantly covering the sample. After saturation, the sample was removed, gently dried from the water (e.g., with a damp cloth), and weighed. Weighing was carried out every 24 h until the change in mass was less than 0.2%. Water absorption was expressed as a percentage increase in the mass of the sample in relation to the mass of the dry sample:

(3) $$N_{w}=\frac{m_{n}-m_{s}}{m_{s}}\times 100\%$$

where *m*_n_—mass of saturated sample (g), *m*_s_—mass of dry sample (g).

*Capillary Uptake.* For these tests, Perlite concrete samples measuring 50 × 40 × 330 mm were cut from ready-made chimney blocks. The samples were then dried at 40 ± 2 °C to constant mass and then cooled to a test temperature of 20 ± 2 °C. Deionised water was used for the tests. The immersion height of the sample was 10 ± 1 mm. The prepared sample was placed on a grid above the water level in such a way that its lower edge was immersed exactly 10 mm in water. Then, at specified time intervals (1, 3, 6, 8 and 24 h), the sample was taken out, water was removed from the surface (e.g., with a paper towel) and weighed to the nearest 0.1 g. The test result was the mass increase in % to the nearest 0.01%.

*Concrete Hydrophobicity Test (“Water Drop Test”)*. The test aimed to assess the hydrophobicity of the perlite concrete surface, and it is mainly used as a quick test of the quality of surface or mass hydrophobicity. The surface of the perlite concrete samples was clean, dry, and free from dust, grease, and efflorescence. The samples were stabilised in laboratory conditions (temperature 20 ± 2 °C and humidity approx. 60%). A pipette applied one drop of water (approx. 20–50 µL) to the tested sample surface. Then, the drop behaviour was observed over time at intervals of 30 s, 1, 4, and 8 h. During the observations, the following were noted: (i) whether the drop retained its spherical shape (high hydrophobicity), (ii) whether it spread (lack of hydrophobicity), and (iii) whether it was absorbed (surface absorbency). The test result is descriptive according to the behaviour of the drop on the surface, i.e.,

- The drop maintains its shape > 5 min → hydrophobic surface;
- The drop spreads but does not soak in → a slightly hydrophobic surface;
- The drop soaks in immediately → non-hydrophobic surface.

The authors attempted to maintain the same manufacturing and curing conditions for each sample in both tests (laboratory and production line) to make the results comparable.

## 3. Test Results of Perlite Concrete and Discussion

### 3.1. Laboratory Sample Testing

**Figure 1:**
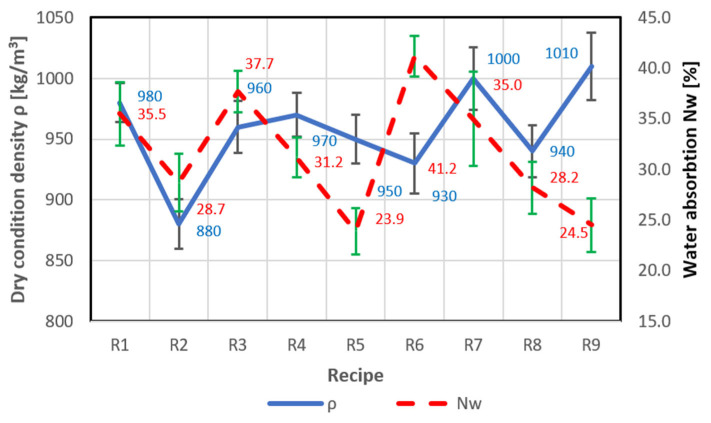
Test results for density and water absorption of mixes (recipes) R1–R9.

**Figure 2:**
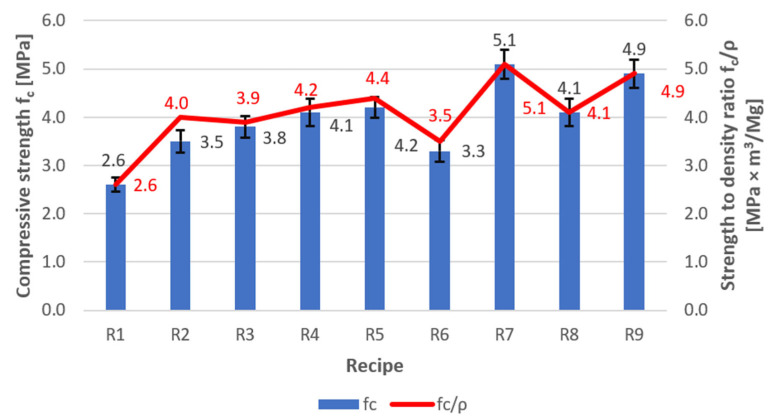
Compressive strength and strength-to-density ratio test results for recipes R1–R9.

Tests on the effect of the type of chemical admixture with a hydrophobic effect on the water absorption of lightweight perlite-based concrete were carried out using samples based on recipe R0, as presented in Table 2. At the same time, considering previous research experience with lightweight concretes and the brittleness of perlite grains, it was decided to determine each concrete sample’s bulk density and compressive strength. Preparations for testing began with producing a reference sample (R1—without chemical admixture with a hydrophobic effect). Subsequent optimisation studies were carried out on perlite concrete mixes containing various chemical admixtures with a hydrophobic effect (recipes R2–R9). The test results for density, water absorption, and compressive strength of recipes R1–R9 are presented in Figure 1 and Figure 2. Table 5 presents the standard deviations for compressive strength, density, and water absorption.

Analysing the test results presented in Figure 1 and Figure 2, it can be stated that the best technical parameters were achieved for mix R5, which exhibited a water absorption of 23.9%, a relatively low concrete density of 950 kg/m^3^, and a reasonably high compressive strength of 4.2 MPa. For mix R9, a low water absorption value of 24.5% and a high compressive strength of 4.9 MPa were obtained; however, the concrete density reached 1010 kg/m^3^, which did not comply with the primary objective of the study—namely, not exceeding 1000 kg/m^3^. The highest compressive strength—5.1 MPa—and the highest strength-to-density ratio (*f*_c_/*ρ* = 5.1) were obtained for mix R7; however, this was accompanied by high water absorption (35%) and a borderline density value of 1000 kg/m^3^. The concrete produced using mix R6 exhibited the lowest density at 930 kg/m^3^, but also the highest water absorption of 41.2% and a compressive strength of only 3.3 MPa, which disqualified this mix (recipe). The test results for mix R1 also did not indicate favourable parameters, i.e., high water absorption of 35.5%, the lowest compressive strength of 2.6 MPa, and the poorest strength-to-density ratio (*f*_c_/*ρ* = 2.6).

**Figure 3:**
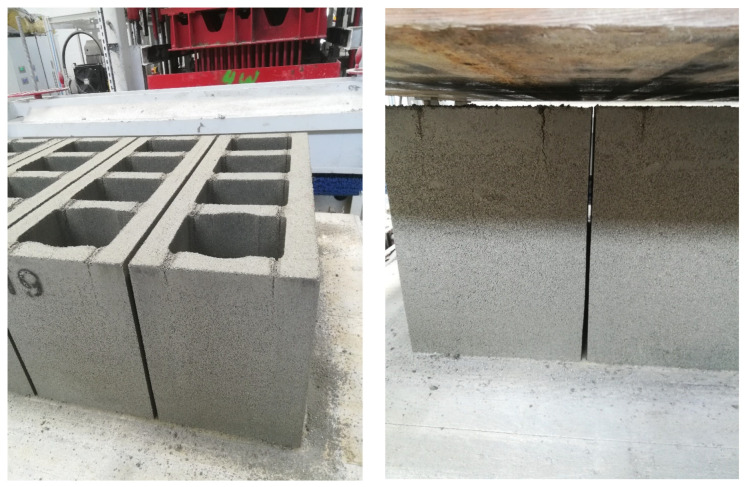
Perlite concrete blocks after demoulding. Visible cracks in the upper sections of the vertical internal and external walls.

Laboratory tests enabled the selection of a perlite concrete mix (R5) that met the assumptions outlined in the introduction. However, attempts to produce chimney blocks on the production line revealed that manufacturing them based on formulations developed under laboratory conditions was less effective than anticipated. The use of laboratory-developed mixes resulted in excessive “waste” during the demoulding of the chimney blocks, which necessitated further trials with different chemical admixtures to eliminate the excessive adhesion of fresh concrete to the mould walls. Many chimney blocks produced using the laboratory-developed formulations exhibited visible wall cracking immediately after demoulding, particularly in the upper section (Figure 3). This is probably due to how the samples are compacted in the laboratory, i.e., the conventional method, which differs from the technology used in production conditions.

Scaling up the research to the production level revealed and addressed challenges absent at the laboratory scale. These findings further suggest a potential direction for extending the scope of investigations described in [8], where adopting a similar experimental framework could provide deeper insights into the reported result.

### 3.2. Production Line Testing

After completing the tests on laboratory-prepared samples, tests were initiated on actual chimney blocks produced on the production line. It was decided to conduct preliminary tests for the developed recipes (R16–R24) to evaluate the effect of moisture exposure on concrete based on expanded perlite, i.e., the drop test based on visual assessment and the capillary water uptake test (by PN-EN 1015-18:2003 [48]). Due to some problems with demoulding chimney blocks on the production line, several additional chemical admixtures for hydrophobising perlite concrete elements (A8–A13—see Table 3 and Table 4) were preliminarily (provisionally) tested. These admixtures were intended to provide both suitable mix consistency and tightness of the hardened concrete.

The initial composition of the perlite concrete mix was adopted based on previous laboratory sample tests (recipe R5). Due to the varying moisture content of the aggregate (moisture coefficient), adjustments to the water content in the mix were made on an ongoing basis to obtain a perlite concrete that could be demoulded effectively on the production line.

Taking into account the conditions on the production line, the following procedure for mixing and dosing individual components was adopted: sand was delivered from feed hoppers onto a scale, from which, after the appropriate quantity was measured, it was transferred to the mixer. Perlite, on the other hand, was measured volumetrically and supplied from a silo. Simultaneously, the amount of cement was measured, and the initial portion of water was added. The aggregates were mixed for approximately 20 s, after which the chemical admixtures were introduced into the mixer and mixed for an additional 5 s. In the next step, cement was added, and the entire batch was mixed for another 20 s. After that, the remaining amount of water was added to achieve the desired moisture content of the mix. The entire mixing process lasted about 1 min. An additional 15 s were required to discharge the concrete mix onto the conveyor belt, which was transported into the moulds.

**Figure 4:**
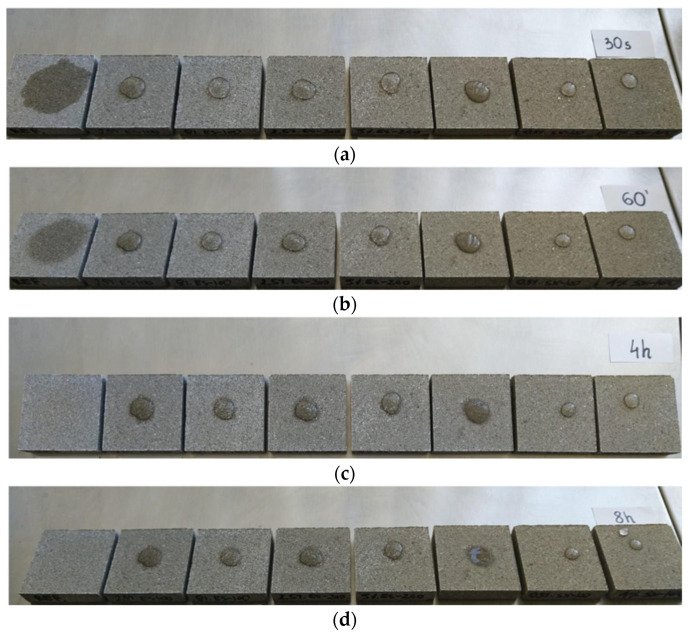
Perlite concrete samples during the “water drop test” at (**a**) 30 s, (**b**) 60 min, (**c**) 4 h, and (**d**) 8 h. From the left: reference sample (mix R16), followed by samples based on mixes R17, R18, R19, R20, R24, R21, and R22.

As a first step, it was decided to assess the water absorption rate by perlite concrete samples R16–R24 using the so-called “water drop test.” If water is quickly absorbed into the surface of the concrete, it indicates higher porosity and, consequently, lower resistance to water and other atmospheric factors. This may suggest that the concrete is less durable and more susceptible to damage, such as that caused by freeze–thaw cycles. This test is primarily used for rapid visual assessment of concrete quality during construction or in laboratories, but it does not provide comprehensive information about all material properties. For this reason, it is mainly used as an indicator of overall surface porosity. A series of samples measuring 9 × 9 × 2 cm was prepared from the mix by pressing with a force of 25 kN (Figure 4).

**Figure 5:**
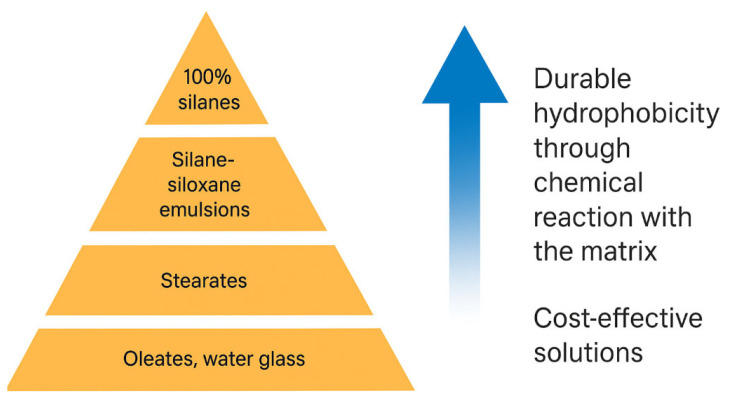
Hierarchy of admixtures reducing capillary uptake—based on raw material composition [49].

A drop of water was applied to each prepared sample, and the shape and absorption into the hardened concrete were observed after 30 s, 1 h, 4 h, and 8 h (Figure 4). As can be seen, water droplets remained on the surface of all samples (except the reference) at the end of the observation period, confirming the effectiveness of the applied chemical admixtures with a hydrophobic effect. The best results were obtained for mixes R21 and R22, in which silane-based admixture A10 (pH 7.0–9.0) was used. The least effective hydrophobisation was observed in mix R24, which contained admixture A11 based on calcium stearate. The effect of the calcium stearate-based admixture on water permeability is clearly visible compared to the reference sample. This is likely due to the calcium stearate forming a wax-like liquid on the capillary surfaces, which limits water absorption. However, there is also a risk of reducing the concrete’s compressive strength by slowing down the hydration process. This effect can be compensated for, for example, by using additives such as fly ash or silica, although this would increase density. The results for the remaining admixtures were significantly better, confirming the hierarchy in Figure 5 [49].

**Figure 6:**
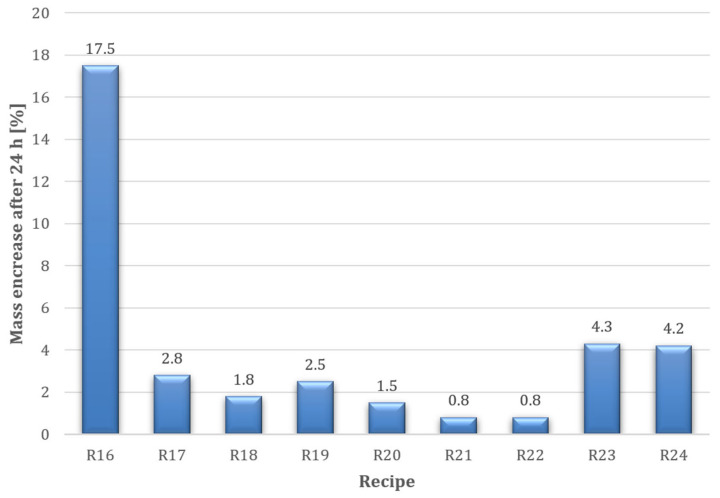
Maximum capillary uptake results of the tested perlite concrete samples (R16–R24) after 24 h.

Capillary uptake tests were also carried out after 7 days of curing. Prepared samples measuring 50 × 40 × 330 mm—cut from the corners of chimney blocks—were placed with their narrowest edge in water (at a depth of 10 mm). The samples were weighed after 1, 3, 6, 8, and 24 h. The tests were conducted using the procedure described in PN-EN 1015-18:2003 [48]. The test results are presented in Figure 6.

The reference sample (recipe R16) results indicate a very high moisture increase caused by capillary water uptake. A clearly beneficial influence on limiting capillary uptake can be observed for samples produced with chemical admixtures with a hydrophobic effect. The lowest mass increase (0.8%) was recorded for samples based on mixes R21 and R22 (with admixture A10 in different dosages). It is evident that there is no difference between the 0.5% and 1.0% dosage of admixture A10, meaning that the desired effect can be achieved with the minimum amount of admixture. The weakest hydrophobisation effect was obtained for admixture A11 (recipes R23 and R24—Figure 6). Thus, similar to the water drop test, admixture A11 produced the least favourable result. This confirms the applied method’s effectiveness for rapid hydrophobisation performance evaluation.

Further tests were conducted on full-scale perlite concrete chimney blocks from an industrial production line, manufactured based on mix designs P0–P5 (Table 4). During the trials, the performance of plasticising admixtures A12 and A13 and sealing admixtures A8, A9, and A10 was evaluated. In the tests performed on blocks and samples cut from finished chimney blocks, mix formulations containing chemical admixture A11 were excluded due to the least favourable results obtained in the “water drop test” and capillary uptake test for mixes R16–R24 produced on the production line. Compressive strength tests were performed on the finished blocks according to the procedure described in [50,51,52]. In contrast, water absorption, water drop, and capillary moisture uptake tests were conducted on samples cut from the previously produced chimney blocks.

**Figure 7:**
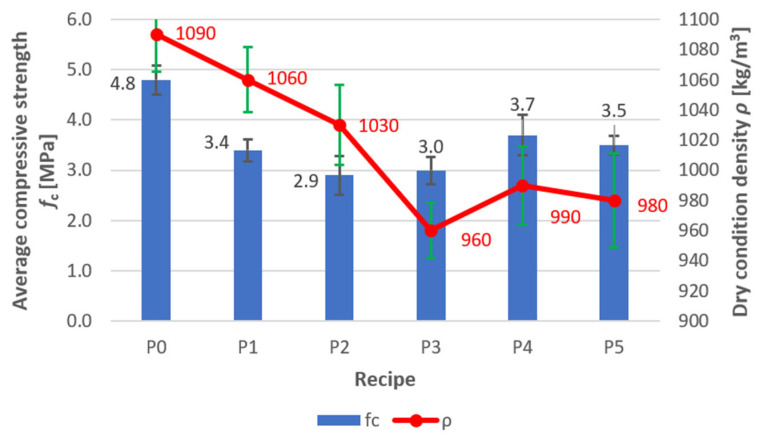
Compressive strength and density of perlite concrete blocks for recipes/mixes P0–P5.

**Figure 8:**
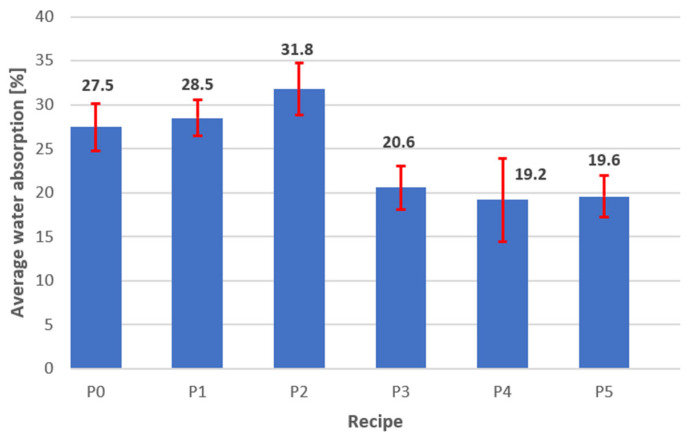
Water absorption of perlite concrete blocks for recipes P0-P5.

**Figure 9:**
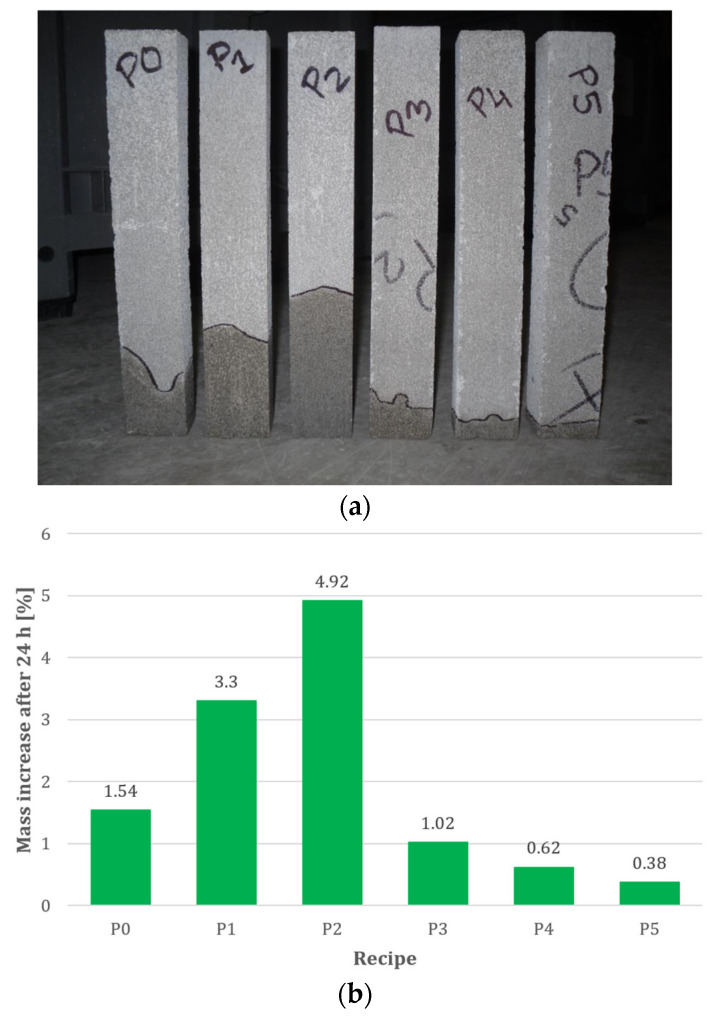
Capillary uptake results of perlite concrete blocks made with mixes P0–P5 after 24 h: (**a**) view of tested samples; (**b**) results after maximum saturation.

**Figure 10:**
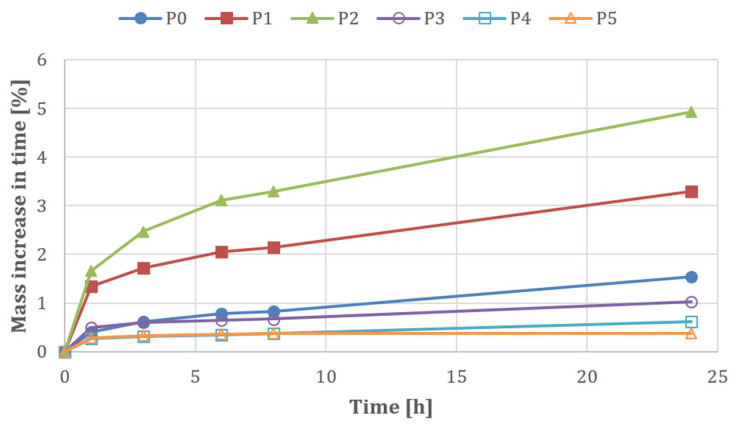
Capillary uptake test results of perlite concrete blocks over time.

**Figure 11:**
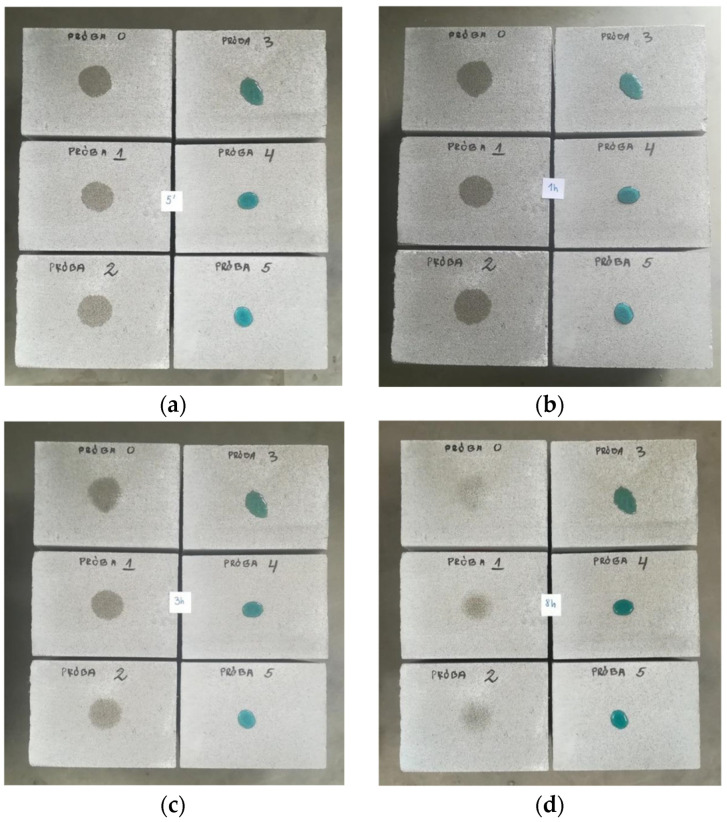
Water drop test results after (**a**) 5 min, (**b**) 1 h, (**c**) 3 h, and (**d**) 8 h. Próba means sample.

The reference mix (recipe) P0 contained the lowest amount of water due to using a superplasticiser based on non-sulfonated acrylic polymers. This is the admixture routinely used on the production line, allowing for a reduction in the amount of mixing water. It was also decided to conduct trials with increased water content (due to the required workability and the need for proper compaction of the perlite concrete blocks). In producing vibropressed concrete products, close control over the moisture content of the concrete in the top layer of the product is crucial. The test results are presented graphically in Figure 7, Figure 8, Figure 9, Figure 10 and Figure 11.

*Compressive Strength and Density.* Figure 7 shows that recipe P0 (reference sample) exhibits the highest compressive strength value (4.8 ± 0.29 MPa), but an excessively high density (1090 ± 24.49 kg/m^3^). Additionally, its relatively high water absorption (27.5 ± 2.69%—Figure 8) and capillary uptake (1.54%—Figure 9) may negatively affect its long-term durability, particularly in environments where the material is exposed to water. Increased water absorption and capillary action can accelerate corrosion processes in structural elements, reducing strength over time.

Using a polymer admixture with increased water retention capacity (recipe P1), which effectively disperses cement grains (improves dispersion), resulted in a nearly 30% reduction in compressive strength. Concrete based on formula P1 achieved a compressive strength of 3.4 ± 0.22 MPa. This change was caused by adding more water to achieve the required consistency. Furthermore, the polymer admixture introduces air bubbles into the mix, facilitating mould filling but further weakening the concrete’s structure.

Using a plasticiser based on aqueous solutions of modified A13 resins further reduced compressive strength (2.9 ± 0.39 MPa achieved—recipe P2). Concrete water absorption also increased. Simultaneously, the uniformity of concrete mix delivery into mould cavities during hollow block production improved, and the cohesion of the compacted concrete mix improved. This reduced the risk of damage during transport to the curing chamber for fresh products. For this reason, it was decided to supplement the recipe with additional hydrophobic admixtures A8–A10 at a rate of 2% by weight of cement. This resulted in concretes with slightly increased compressive strength for recipes P3–P5 (Figure 7).

*Water Absorption*. It can be observed that mix P2, in addition to exhibiting the lowest compressive strength—2.9 ± 0.39 MPa (Figure 7)—also shows high density (1030 ± 26.46 kg/m^3^—Figure 7), the highest water absorption (31.8 ± 3.00%—Figure 8), and capillary uptake (4.92%—Figure 9). This indicates that this mix, aside from its relatively low compressive strength, is the most susceptible to water absorption. This was to be expected, as the A13 admixture has both plasticising and air-entraining properties, which are desirable for streamlining the production of perlite concrete blocks (forming). This increases the risk of material degradation due to moisture and accelerates corrosion processes, especially in the presence of metallic components within the structure. Therefore, since P2 has high water absorption, its use in humid conditions is not recommended, as it is more likely to lose its mechanical properties and durability over time.

A13 is a plasticising and air-entraining admixture based on an aqueous solution of modified resins, while A8–A10 are hydrophobic admixtures based on an emulsion of silanes and siloxanes. These two types of admixtures have different effects. The former is a rheological admixture, while the latter is a hydrophobic admixture. Silanes and siloxanes exhibit strong hydrophobic properties—they can hinder the adsorption of other compounds on the surface of cement grains or aggregates. They can also limit the dispersion of resin components in the A13 admixture. Siloxanes can modify the porous structure by reacting with the C-S-H phase, which can affect the effectiveness of the air-entraining effect. For these reasons, tests were necessary to rule out the risk of admixture incompatibility. After several trials, it was determined that the order in which the ingredients were added was important. Adding the plasticising and air-entraining admixture (A13) first maintained the plasticising effect while maintaining proper hydrophobicity (see water absorption results for recipes P3–P5, Figure 8).

*Capillary Uptake.* Like water absorption, capillary uptake is an indicator that significantly affects material durability. Mix P2 exhibits a high tendency for water uptake (4.92%—Figure 9), resulting in a substantial increase in mass over a short period (24 h). Such properties can lead to material degradation through water penetration and the formation of cracks. This is a result of the loosening of the perlite concrete structure due to the action of the plasticising and air-entraining admixture A13. In contrast, mix P5, although it achieves a compressive strength of 3.5 MPa (which is slightly lower than that of P0—Figure 7), shows appropriate density (980 kg/m^3^—Figure 7), very low water absorption (19.6%—Figure 8), and capillary uptake (0.38%—Figure 9), making it more resistant to the adverse effects of moisture and corrosion processes. This material will retain its mechanical properties and durability for longer, even under more demanding conditions. Furthermore, Figure 10 shows that mixes P1 and P2 exhibit the most significant mass increase within the first hours, confirming that they are most susceptible to water absorption (no hydrophobic admixtures). Mixes P4 and P5 demonstrate significantly lower mass gain, indicating better resistance to capillary water uptake—especially P5, which shows virtually no change in mass. This also demonstrates that the adopted order of component dosing (adding the A13 admixture first, and then adding the silanes/siloxanes (A8–A10) at the end of the mixing process) ruled out the possibility of incompatibility between the two types of chemical admixtures.

As shown in Figure 11, samples P0–P2 exhibit the lowest effectiveness of hydrophobisation—after 8 h, the perlite concrete samples almost completely absorbed the water droplet. This indicates that adding plasticising admixtures A12 (based on a polymer solution) and A13 (based on an aqueous solution of modified resins) at a dosage of 0.5% does not produce the desired hydrophobic effect. In contrast, clear hydrophobic properties are observed for perlite concrete samples P4 and P5, in which admixtures A9 and A10 were used at 2%, respectively—both based on silane/siloxane water emulsions (of different pH).

In summary, it can be concluded that mix P0, despite having the highest compressive strength, exhibits relatively high density, water absorption, and capillary uptake, which may accelerate corrosion processes and weaken its mechanical properties over time. Therefore, its application in moisture-exposed environments should be considered with caution. Mixes P1 and P2, on the other hand, have relatively high density and the highest water absorption and capillary uptake, meaning that despite their relatively high compressive strength, their durability and resistance to moisture are significantly lower. These properties make them the least suitable for long-term use in humid conditions.

In contrast, mixes P4 and P5, although they exhibit slightly lower compressive strength compared to the reference mix P0 (due to the use of silane/siloxane-based chemical additives), show the best resistance to water absorption (in terms of both total water absorption and capillary uptake) while maintaining the required density. This makes them the most durable options in terms of resistance to moisture and corrosion. They are, therefore, the best choice for long-term applications where water exposure is a critical factor.

**Figure 12:**
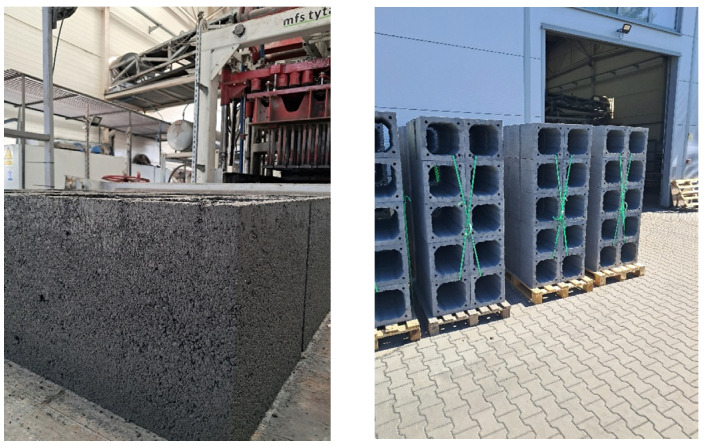
Finished perlite concrete chimney blocks from the production line.

Thus, if the main objective is to ensure durability and moisture resistance at relatively low density, mixes P4 and P5 appear to be the most advantageous (Figure 12), even though their compressive strength is not the highest. P0, P1, P2, and P3 may be used, but only in areas where water resistance is not a key requirement.

Comparing the obtained research results with other studies that concerned the use of lightweight aggregates, such as expanded perlite, expanded clay aggregate and foamed geo-polymer aggregate [8], for the production of chimney casings, it can be concluded that the perlite-concrete block developed in this study, based only on expanded perlite, is characterised by lower density, but higher water absorption and lower compressive strength. However, it should be noted that the obtained compressive strength of perlite concrete at the level of 3.5 MPa and water absorption of 25% are sufficient for chimney blocks, as there is no practical justification for a higher compressive strength and lower water absorption. In the study of lightweight concretes [53], in which pumice, diatomite, and expanded glass were used instead of sand, even higher compressive strengths were obtained than in [8], but also significantly higher densities than in the present study. Furthermore, for example, in study [8] a mixture of various lightweight aggregates was used, i.e., foamed geopolymer aggregate (ca. 51%), expanded clay aggregate (ca. 13%), and a small amount of expanded perlite (0.1%) with a high share of sand and fly ash (ca. 18% each). This resulted in significant compressive strength (approx. 18 MPa) and water absorption (6.7%). Comparison with concrete based on expanded clay aggregate [46] shows that the developed perlite concrete is characterised by similar parameters, with an advantage in terms of density and thermal conductivity. However, compared to typical ceramic [44] and concrete [45] blocks, it achieves better parameters in terms of density, thermal conductivity, and capillary uptake. The results obtained can also be compared to those of the study [36], which involved using 100% expanded perlite instead of sand. In this case, the obtained compressive strength was lower than in this study. In study [35], perlite and marble waste were used to develop various concrete mixtures. However, the obtained density exceeded 1065 kg/m^3^, but adding marble positively affected the increase in compressive strength (min. 8.6 MPa after 28 days of curing). From the above, it is clear that many research studies use expanded perlite and other lightweight aggregates to develop lightweight concretes. However, the most crucial element is defining a given concrete material’s intended use, which determines the requirements for minimum technical parameters. Different technical parameters are expected for chimney casings, different ones for building walls and still different ones for ceilings.

## 4. Conclusions

Based on the laboratory tests and tests on chimney blocks formed on the production line using perlite concrete, the following final conclusions can be drawn:

1. The laboratory test results of samples R1–R9 with various admixtures A1–A7 enabled the design and production of perlite concrete mix R5, which exhibited appropriate parameters regarding density, water absorption, and compressive strength. However, production tests on full-size chimney blocks based on the proposed mix proved to be technically inefficient (a high number of defective blocks were produced during demoulding, and visible cracking was observed). This may be attributed to the compaction method used in the laboratory, which differs from the technology applied under production conditions.
2. The conducted tests on full-scale perlite concrete chimney blocks produced using mixes P0–P5 with various admixtures A8–A10 and A12–A13 led to the development of mix formulations P4 and P5 that met the specified requirements for density, water absorption, capillary uptake, and compressive strength. These performance parameters were achieved using chemical admixtures with a hydrophobic effect based on silane water emulsions. Moreover, the water drop tests confirmed the positive results obtained for mixes P4 and P5. Therefore, the currently applicable standards for chimney casings should be extended to include water absorption testing, at a minimum, using the water drop test method.
3. From a practical standpoint, using chemical admixtures with a hydrophobic effect significantly altered the water demand of the mix and the water-to-cement (*w*/*c*) ratio. As a result, the perlite concrete mix became somewhat less workable, and numerous cracks appeared already during the forming stage, which then propagated. This led to a considerable increase in production waste. To mitigate this effect, additional moisture optimisation of the mix was implemented; however, most importantly, the technology required strict post-forming curing of the products. The manufacturer constructed an experimental curing chamber to ensure optimal concrete maturation conditions and minimise the risk of cracking. With electronically controlled water and ventilation systems, the required temperature and air humidity are maintained during the curing of the prefabricated elements. The curing time was also extended from 3 to 7 days. As a result, the desired production outcomes were achieved: a 25% improvement in block strength after curing in the newly built chamber and an average weight reduction of 10%. The structure of the block became more homogeneous—with no visible cracks.

The research and conclusions may serve as a basis for developing a subsequent, more advanced product—safe chimney penetrations through wooden horizontal and vertical building partitions (ceilings and roofs). These penetrations will be made of perlite concrete with higher insulation properties than those described in this article, to provide fire protection for the wooden elements of the building. Moreover, the obtained results indicate the possibility of using this material in other building elements made of perlite concrete (e.g., wall fillings, ventilation blocks), but this requires additional research.

## Figures and Tables

**Table 1:** Comparison of materials used for porous chimney blocks.

Type of Material	Density (kg/m^3^)	Thermal Conductivity (W/(m·K))	Compressive Strength (MPa)	Water Absorption (%)
Ceramic block [44]	1500	0.4	5	22
Concrete block [45]	2100	1.28	7	6
Leca concrete block [46]	1042	0.45	3.5	16
Perlite concrete block	1000	0.37	3.5	25

**Table 2:** Base Perlite Concrete Mix R0.

Component	Unit	Amount
Perlite	kg per batch	120
Cement I 52.5 R		240
Sand 0–2 mm		330
Water		130
Superplasticiser S1		2.9 (1.2%)
Hydrophobic admixture A1		2.4 (1%)

**Table 3:** Compositions of tested perlite concrete mixes R16–R24 based on recipe R0.

Component	Unit	Recipe								
		R16	R17	R18	R19	R20	R21	R22	R23	R24
Perlite	m^3^	1.2								
Cement CEM I 42.5 R	kg	240								
Sand 0–2 mm	kg	330								
Water	L	160								
A8	%	-	2.5	5	-	-	-	-	-	-
A9	%	-	-	-	2.5	5	-	-	-	-
A10	%	-	-	-	-	-	0.5	1	-	-
A11	%	-	-	-	-	-	-	-	0.5	1

**Table 4:** Detailed description of mix formulations P0–P5 used for the production of perlite concrete blocks for testing (products from the production line).

Component	Unit	Recipe					
		P0	P1	P2	P3	P4	P5
Perlite	m^3^	1.2					
Cement CEM I 42.5 R	kg	240					
Sand 0–2 mm	kg	330					
Water	L	156	184	175	170	170	173
S1	kg	2.9	-	-	-	-	-
A8	%	-	-	-	2.0	-	-
A9	%	-	-	-	-	2.0	-
A10	%	-	-	-	-	-	2.0
A12	%	-	0.5	-	-	-	-
A13	%	-	-	0.5	0.5	0.5	0.5

**Table 5:** Summary of standard deviations for compressive strength, density, and water absorption.

Recipe	Compressive Strength	Standard Deviation	Density	Standard Deviation	Water Absorption	Standard Deviation
-	MPa	-	kg/m^3^	-	%	-
R1	2.6	0.15	980	16.26	35.5	3.15
R2	3.5	0.23	880	20.20	28.7	2.88
R3	3.8	0.23	960	21.60	37.7	2.02
R4	4.1	0.28	970	18.26	31.2	2.00
R5	4.2	0.21	950	20.28	23.9	2.29
R6	3.3	0.22	930	24.49	41.2	2.00
R7	5.1	0.30	1000	25.82	35.0	4.67
R8	4.1	0.28	940	21.60	28.2	2.58
R9	4.9	0.29	1010	27.69	24.5	2.66
P0	4.8	0.29	1090	24.49	27.5	2.69
P1	3.4	0.22	1060	21.60	28.5	2.06
P2	2.9	0.39	1030	26.46	31.8	3.00
P3	3.0	0.27	960	18.26	20.6	2.47
P4	3.7	0.40	990	25.82	19.2	4.70
P5	3.5	0.18	980	31.09	19.6	2.40

## Data Availability

The original contributions presented in this study are included in the article. Further inquiries can be directed to the corresponding author.
